# Gremlin-1 and BMP-4 Overexpressed in Osteoarthritis Drive an Osteochondral-Remodeling Program in Osteoblasts and Hypertrophic Chondrocytes

**DOI:** 10.3390/ijms23042084

**Published:** 2022-02-14

**Authors:** Maria-Luisa Pérez-Lozano, Laure Sudre, Sandy van Eegher, Danièle Citadelle, Audrey Pigenet, Marie-Helène Lafage-Proust, Philippe Pastoureau, Frédéric De Ceuninck, Francis Berenbaum, Xavier Houard

**Affiliations:** 1 Centre de Recherche Saint-Antoine (CRSA), INSERM, Sorbonne Université, F-75012 Paris, France; marialuisa.perezlozano@univ-paris13.fr (M.-L.P.-L.); laurette_s@yahoo.com (L.S.); sandy.vaneegher@gmail.com (S.v.E.); daniele.citadelle@upmc.fr (D.C.); audrey.pigenet@sorbonne-universite.fr (A.P.); xavier.houard@sorbonne-universite.fr (X.H.); 2 Faculté de Médecine, INSERM U1059, Université Jean Monnet, F-42270 Saint-Priest en Jarez, France; mh.lafage.proust@univ-st-etienne.fr; 3 Immuno-Inflammatory Diseases Department, Servier Research Institute, F-92150 Suresnes, France; pastouph@yahoo.fr (P.P.); frederci.deceuninck@servier.com (F.D.C.); 4 Centre de Recherche Saint-Antoine (CRSA), INSERM, Sorbonne Université, AP-HP Hôpital Saint Antoine, F-75012 Paris, France

**Keywords:** gremlin-1, Bmp-4, osteochondral remodeling, mechanical stress, osteoarthritis, hypertrophic chondrocytes

## Abstract

Osteoarthritis (OA) is a whole joint disease characterized by an important remodeling of the osteochondral junction. It includes cartilage mineralization due to chondrocyte hypertrophic differentiation and bone sclerosis. Here, we investigated whether gremlin-1 (Grem-1) and its BMP partners could be involved in the remodeling events of the osteochondral junction in OA. We found that Grem-1, BMP-2, and BMP-4 immunostaining was detected in chondrocytes from the deep layer of cartilage and in subchondral bone of knee OA patients, and was positively correlated with cartilage damage. ELISA assays showed that bone released more Grem-1 and BMP-4 than cartilage, which released more BMP-2. In vitro experiments evidenced that compression stimulated the expression and the release of Grem-1 and BMP-4 by osteoblasts. Grem-1 was also overexpressed during the prehypertrophic to hypertrophic differentiation of murine articular chondrocytes. Recombinant Grem-1 stimulated Mmp-3 and Mmp-13 expression in murine chondrocytes and osteoblasts, whereas recombinant BMP-4 stimulated the expression of genes associated with angiogenesis (Angptl4 and osteoclastogenesis (Rankl and Ccl2). In conclusion, Grem-1 and BMP-4, whose expression at the osteochondral junction increased with OA progression, may favor the pathological remodeling of the osteochondral junction by inducing a catabolic and tissue remodeling program in hypertrophic chondrocytes and osteoblasts.

## 1. Introduction

Osteoarthritis (OA) is the most common musculoskeletal degenerative disease and a major cause of pain and disability in the elderly [1,2,3,4]. The main risk factors for OA include age, obesity, and joint injury [5]. OA is a whole joint disease, characterized by the irreversible degradation of cartilage, the pathological remodeling of the subchondral bone, including sclerosis and osteophytosis as well as to moderate inflammation of the synovium [2,6,7]. The remodeling of the osteochondral junction is thought to play a determinant role in OA and is reminiscent of the endochondral ossification process. It involves the hypertrophic differentiation of chondrocytes of the deepest layer of the hyaline articular cartilage followed by the mineralization and the vascularization of the cartilage matrix and the replacement of cartilage by bone, leading to sclerosis of the subchondral bone plate [8,9,10]. This complex process requires strong functional interactions between chondrocytes and bone cells. However, mechanisms and biochemical mediators governing the remodeling of the osteochondral junction in OA remain poorly characterized.

Mechanical loading is determinant for maintaining the integrity of bone, skeletal mass, and architecture. It is well established that mechanical loading enhances bone formation and favors cartilage homeostasis, whereas unloading leads to bone loss and cartilage degradation [11,12,13,14,15,16]. However, inappropriate mechanical loading, as occurs with an excessive body weight, weakness of muscles related to aging or instability after a ligament injury, promotes cartilage degradation and subchondral bone remodeling, leading to the development of OA [7,11,12,17,18,19]. Osteoblasts, osteocytes, and chondrocytes are all mechanosensitive cells that release catabolic and inflammatory factors when submitted to an excessive mechanical load [20,21,22,23,24]. Moreover, compression was proposed to be responsible for the phenotypic changes of osteoblasts in OA subchondral bone leading to sclerosis of the subchondral bone plate [22]. In this context, Gremlin-1 (Grem-1) has been identified as a mechanosensitive factor, whose expression is increased in articular cartilage under a tensile mechanical loading or joint injury [25,26,27,28]. The expression of Grem-1 in articular cartilage and its concentration in the serum of OA patients have been correlated with OA progression [14,26,29,30]. Interestingly, Grem-1 expression in OA cartilage is increased in the deepest layers of cartilage, where hypertrophic chondrocytes are found [28]. The hypertrophic differentiation of chondrocytes is a typical feature of OA cartilage and is currently considered as playing a pivotal role in cartilage disappearance and subchondral bone remodeling [10].

Grem-1 acts through VEGFR-2 activation or bone morphogenetic protein (BMP) antagonism [31,32,33,34]. BMPs play an important role in skeletal tissue development and homeostasis. BMP-2, BMP-4, and BMP-7, which can be targeted by Grem-1 [30,35,36,37], indeed stimulate chondrocyte proliferation and extracellular matrix production and show joint protective effects. However, their expression increases with age-associated cartilage degradation in the temporomandibular joint [38]. BMP-2 is detected in OA cartilage, but it is expressed at a low level in healthy cartilage [39]. Interestingly, BMP-2, present in the cartilage and the bone of osteophytes [39], has been involved in osteophyte development, a typical feature of the pathological bone remodeling in OA [40]. In addition, serum levels of BMP-2 are increased in patients with hip OA, as it is also observed for BMP-4 [41]. However, the role of BMP-4 in OA is less characterized.

In the present study, we investigated the expression of Grem-1 and its partners at the osteochondral junction of patients with knee OA and by compressed osteoblasts and chondrocytes during their hypertrophic differentiation. Finally, we investigated the response of osteoblasts, prehypertrophic, and hypertrophic chondrocytes to Grem-1 and BMP-4 stimulation. Our results suggest that Grem-1 and BMP-4 may act as partners in the remodeling events occurring at the osteochondral junction in OA.

## 2. Results

### 2.1. Overexpression of Gremlin-1 and BMPs at the Osteochondral Junction with OA Progression

**Figure 1:**
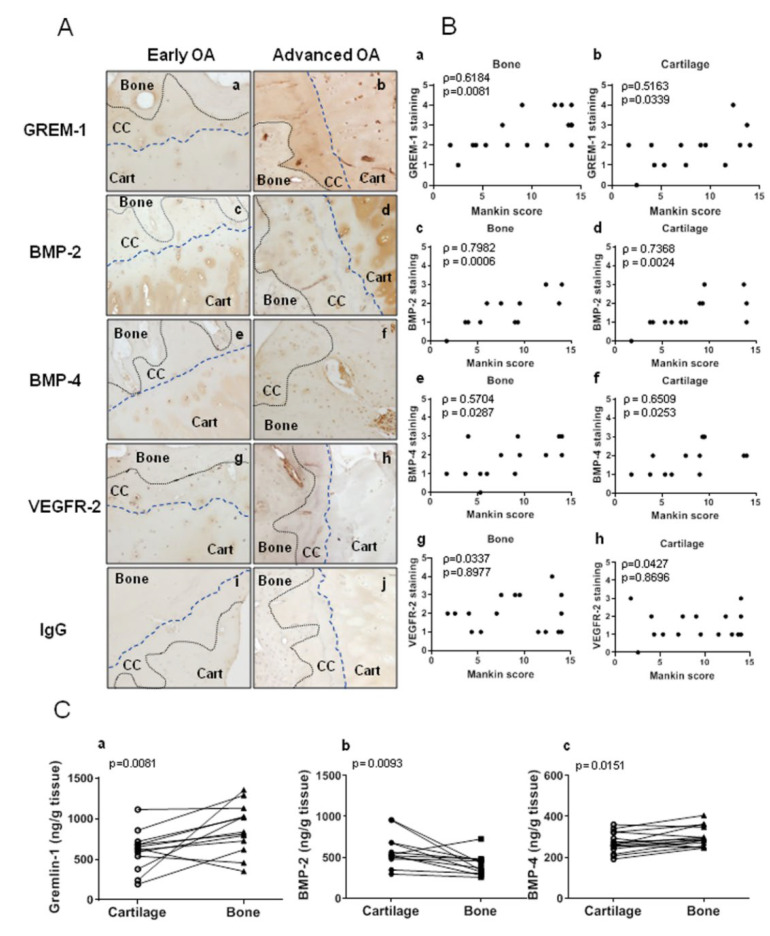
Gremlin-1 and BMPs expression in human osteochondral junction during OA progression. Panel (**A**) Immunohistochemical staining of Gremlin-1 (**a**,**b**), BMP-2 (**c**,**d**), BMP-4 (**e**,**f**), VEGFR-2 (**g**,**h**), and control IgG (**i**,**j**) in 5 μm paraffin sections of human OA cartilage-bone interface. Cartilage and bone are delimited by black dotted lines, and cartilage and calcified cartilage are delimited by blue dotted lines. Cart: cartilage, CC: calcified cartilage. Scale bars = 50 μm. Pictures are representative of 17 tissue samples from 6 OA patients. Panel (**B**) Correlations between OA score evaluated by the Mankin score and the expression of Grem-1 (**a**,**b**), BMP-2 (**c**,**d**), BMP-4 (**e**,**f**), VEGFR-2 (**g**,**h**) in bone (**a**,**c**,**e**,**g**) and cartilage (**b**,**d**,**f**,**h**) (n = 15–17). (**C**) Release of Grem-1 (**a**) (n = 17), BMP-2 (**b**) (n = 15) and BMP-4 (**c**) (n = 15) by bone and cartilage from OA patients. The lines link cartilage and bone samples from the same patients.

Both human OA cartilage and bone released Grem-1 in tissue-conditioned media with higher concentration found in bone (879.7 ± 83.2 ng/g tissue) than in cartilage supernatants (608.01 ± 67.4 ng/g tissue) (*p* = 0.0081) (Figure 1(Ca)). A Grem-1 positive staining was associated with chondrocytes in non-calcified and calcified cartilage, with osteoblasts and osteocytes, and with vessels and mesenchymal cells within vascular channels in the subchondral bone (Figure 1(Aa,Ab)). Interestingly, a more intense staining can be observed in advanced OA tissues as compared to early OA, both in cartilage and subchondral bone. Moreover, a positive correlation between cartilage damage and Grem-1 positive immunostaining was found in cartilage (ρ = 0.5163, *p* = 0.0339) and bone (ρ = 0.6184, *p* = 0.008) (Figure 1(Ba,Bb)). As for Grem-1, BMP-2 and BMP-4 were released by human OA cartilage and bone. The concentration of BMP-4 in the conditioned media of bone was higher than that of cartilage (1.2-fold, *p* = 0.0151). In contrast, the release of BMP-2 by cartilage was higher than by bone (1.4-fold, *p* = 0.0093) (Figure 1(Cb,Cc)). Consistently, BMP-2 immunostaining was mainly observed in the cartilage and BMP-4 in the subchondral bone, where the staining was observed in osteocytes and in vascular channels (Figure 1(Ac–Af)). It is noteworthy that their expression in cartilage and subchondral bone appeared stronger in advanced OA, as compared to early OA and they were positively correlated, in both cartilage and subchondral, to the progression of OA evaluated by the Mankin score (ρ = 0.7368, *p* = 0.0024 and ρ = 0.7982, *p* = 0.0006 for BMP-2 in cartilage and subchondral bone, respectively and ρ = 0.6509, *p* = 0.0253 and ρ = 0.631, *p* = 0.0138 for BMP-4 in cartilage and subchondral bone, respectively) (Figure 1(Ac–Af,Bc–Bf)). Positive immunostaining for VEGFR-2 and BMPRs was also detected in cartilage and subchondral bone (Figure 1A and Supplementary Figure S1A). Whereas the expression of BMPRs increased with OA progression and showed significant positive correlations for BMPR1a and BMPR1b with the Mankin score, no variation of VEGR2 and BMPR-2 was observed according to the OA grade (Figure 1A and Supplementary Figure S1A).

### 2.2. Gremlin-1 Expression and Release Increased under Mechanical Compressive Loading on Osteoblasts

**Figure 2:**
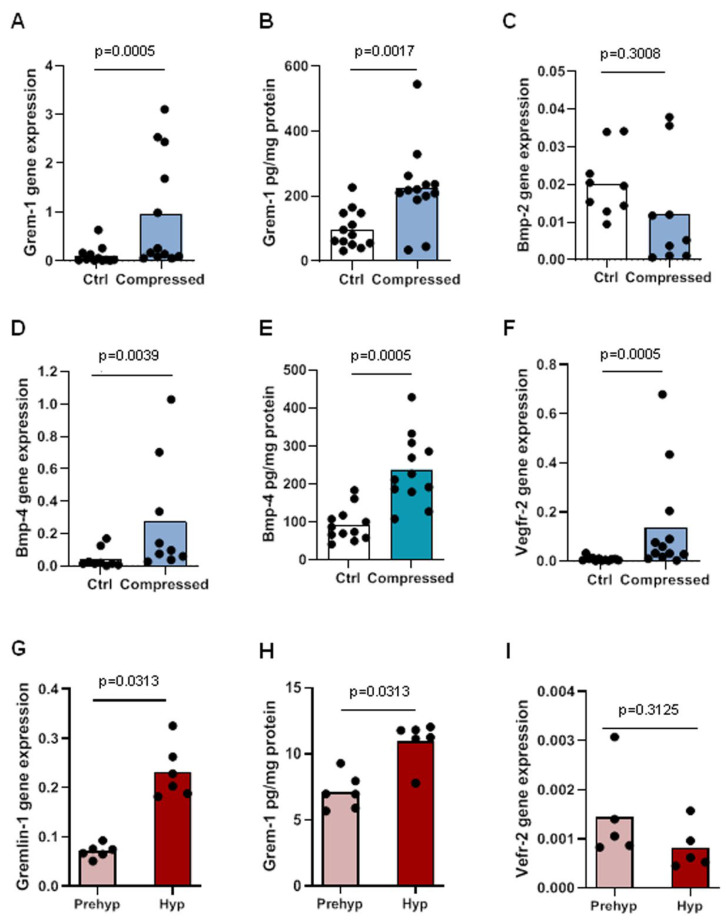
Expression of Gremlin-1 and its partners in compressed osteoblasts and in prehypertrophic and hypertrophic chondrocytes. mRNA expression of Grem-1 (**A**), Bmp-2 (**C**), Bmp-4 (**D**), and Vegfr-2 (**F**) in compressed and control osteoblasts (n = 9–12) and of Grem-1 (**G**) and VEGFR-2 (**I**) in prehypertrophic and hypertrophic chondrocytes (n = 6) was determined. Grem-1 concentration was measured in cell-conditioned media of compressed and control osteoblasts (**B**) and of prehypertrophic and hypertrophic chondrocytes (**H**). BMP-4 released was measured in cell-conditioned media of compressed and control osteoblasts (**E**). Bars indicate the mean expression levels. Ctrl: control, Prehyp: prehypertrophic, Hyp: hypertrophic.

Phenotypic alterations of chondrocytes and osteoblasts characterize OA cartilage and subchondral bone, including the differentiation of articular chondrocytes into hypertrophic cells and the acquisition of specific features by osteoblasts, which can be mimicked in vitro by excessive mechanical load [21,22]. The expression of Grem-1 and its partners was investigated in murine osteoblasts after prolonged mechanical stress and in murine articular chondrocytes undergoing hypertrophic differentiation. Mechanical stress induced a strong upregulation of Grem-1 mRNA expression (8.5-fold, *p* = 0.0005) in osteoblasts (Figure 2A). The release of Grem-1 was also higher when osteoblasts were compressed (2.3-fold, *p* = 0.0017) (Figure 2B). Excessive compressive loading also up-regulated Bmp-4 mRNA expression (6.3-fold, *p* = 0.0039) and BMP-4 secretion (2.5-fold, *p* = 0.0005) in compressed osteoblasts compared to control but had little effect on Bmp-2 mRNA expression (1.6-fold, *p* = 0.3) (Figure 2C–E). A strong increase in Vegfr-2 (16.6-fold, *p* = 0.0005) and Bmpr-1b (6.3-fold, *p* = 0.0039) mRNA expression was induced by the compression, whereas Bmpr-1a expression (2.3-fold, *p* = 0.039) was downregulated (Figure 2F and Supplementary Figure S1(Ca,Cb)). The mRNA expression of Bmpr-2 did not show any statistical differences (*p* = 0.57) (Supplementary Figure S1(Cc)).

We previously showed in a model of progressive hypertrophic differentiation of murine articular chondrocytes that the prehypertrophic to hypertrophic differentiation was associated with an overexpression of both Bmp-2 and Bmp-4 Mrna [42]. Similarly, Grem-1 mRNA expression (3.2-fold, *p* = 0.0313) and Grem-1 secretion (1.5-fold, *p* = 0.0313) increased with the hypertrophic differentiation of prehypertrophic chondrocytes (Figure 2G,H). In contrast, neither the mRNA expression of VEGFR2 nor that of BMPRs was modified by the phenotypic transition of prehypertrophic into hypertrophic chondrocytes (Figure 2I and Supplementary Figure S1(Cd–Cf)).

Together, these results show that the mechanical loading of osteoblasts and the hypertrophic differentiation of prehypertrophic chondrocytes are both associated with an overexpression of GREM-1 and BMP-4, suggesting that their interplay may play a role in the remodeling of the osteochondral junction in OA.

### 2.3. Grem-1 and BMP-4 Increase the Remodeling Potential of Osteoblasts and Chondrocytes

The main cells of the osteochondral junction include osteoblasts/osteocytes in the subchondral bone, prehypertrophic and hypertrophic chondrocytes in the deepest part of the non-calcified cartilage and in the calcified cartilage. Murine osteoblasts, prehypertrophic, and hypertrophic chondrocytes were stimulated with recombinant mouse Grem-1 (rmGREM-1) and BMP-4 (rmBMP-4), alone or in combination, to determine their effect on the tissue remodeling potential of osteoblasts and chondrocytes.

**Figure 3:**
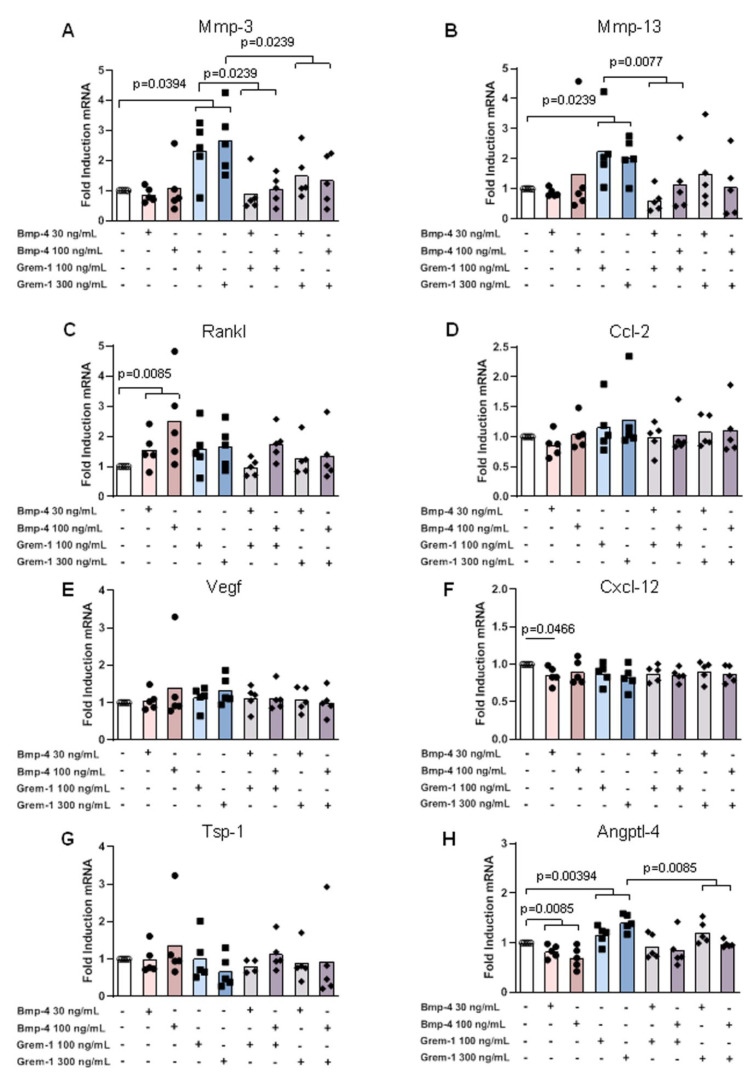
Effects of rmGremlin-1 and rmBMP-4 in osteoblasts. Osteoblasts (n = 5) were stimulated by increasing concentrations of rmBMP-4 (30 and 100 ng/mL) and rmGREM-1 (100 and 300 ng/mL), alone and in combination for 24 h. The mRNA expression of Mmp3 (**A**), Mmp13 (**B**), Rankl (**C**), Ccl-2 (**D**), Vegf (**E**), Cxcl-12 (**F**), Tsp-1 (**G**), and Angptl-4 (**H**) was analyzed by RT-qPCR. Bars indicate the mean expression levels.

**Figure 4:**
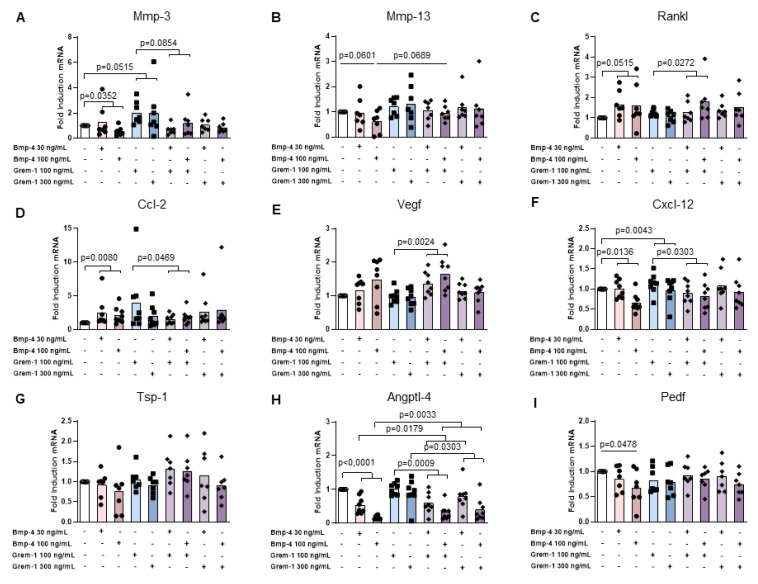
Effects of rmGremlin-1 and rmBMP-4 in prehypertrophic chondrocytes. Prehypertrophic chondrocytes (n = 8) were stimulated by increasing concentrations of rmBMP-4 (30 and 100 ng/mL) and rmGREM-1 (100 and 300 ng/mL), alone and in combination for 24 h. The mRNA expression of Mmp3 (**A**), Mmp13 (**B**), Rankl (**C**), Ccl-2 (**D**), Vegf (**E**), Cxcl-12 (**F**), Tsp-1 (**G**), Angptl-4 (**H**), and Pedf (**I**) was analyzed by RT-qPCR. Bars indicate the mean expression levels.

**Figure 5:**
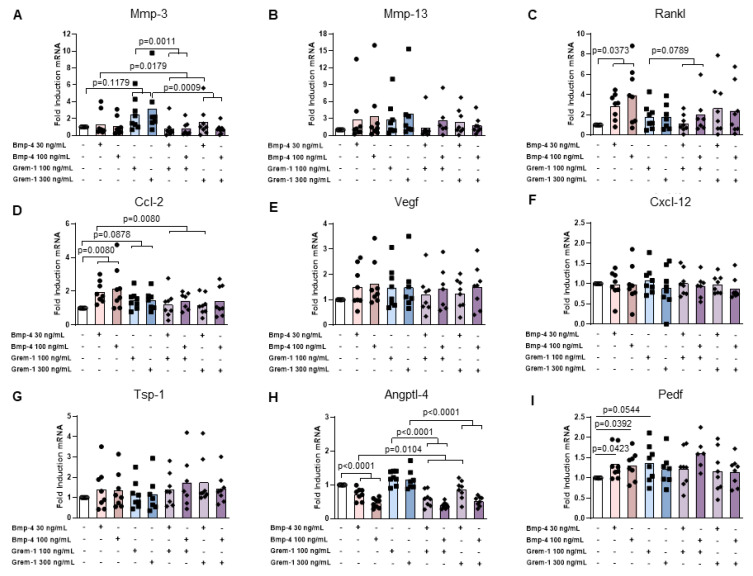
Effects of rmGremlin-1 and rmBMP-4 in hypertrophic chondrocytes. Hypertrophic chondrocytes (n = 8) were stimulated by increasing the concentrations of rmBMP-4 (30 and 100 ng/mL) and rmGREM-1 (100 and 300 ng/mL), alone and in combination during 24 h. The mRNA expression of Mmp3 (**A**), Mmp13 (**B**), Rankl (**C**), Ccl-2 (**D**), Vegf (**E**), Cxcl-12 (**F**), Tsp-1 (**G**), Angptl-4 (**H**), and Pedf (**I**) was analyzed by RT-qPCR. Bars indicate the mean expression levels.

rmGREM-1 significantly stimulated the mRNA expression of metalloproteinases Mmp-3 (2.5-fold, *p* = 0.0394) and Mmp-13 (2.2-fold, *p* = 0.0239) and the angiostatic factor Angptl-4 (1.4-fold, *p* = 0.0394) in osteoblasts, whereas no effect could be observed for other factors involved in osteoclast function and angiogenesis (Figure 3A–H). Similarly, an upward trend for increased expression of Mmp-3 (2.0- and 3.0-fold respectively) was observed in prehypertrophic and hypertrophic chondrocytes in response to rmGREM-1, although it did not reach statistical significance (Figure 4A and Figure 5A). In addition, the mRNA expression of Cxcl-12 was also stimulated by rmGREM-1 in prehypertrophic chondrocytes (1.2-fold, *p* = 0.0443) (Figure 4F) and that one of Ccl-2 tended to be in hypertrophic chondrocytes (1.4-fold, *p* = 0.0878) (Figure 5D). No effect of rmGREM-1 could be observed on prehypertrophic and hypertrophic chondrocytes for other genes studied.

rmBMP-4 stimulated the mRNA expression of the osteoclastogenic factor Rankl in osteoblasts (2.5-fold, *p* = 0.0085), prehypertrophic (1.6-fold, *p* = 0.0515), and hypertrophic chondrocytes (4-fold, *p* = 0.0373), whereas it decreased that of Angptl-4 (1.4-fold, *p* = 0.0085; 5.8-fold, *p* < 0.0001 and 2.2-fold, *p* < 0.0001, for osteoblasts, prehypertrophic and hypertrophic chondrocytes, respectively) (Figure 3C–H, Figure 4C–H and Figure 5C–H). While rmBMP-4 did not induce any other effects in osteoblasts, it stimulated the expression of Ccl-2 in both prehypertrophic and hypertrophic chondrocytes (2.4-fold, *p* = 0.0080 and 2.1-fold, *p* = 0.0080) (Figure 4D and Figure 5D) and downregulated that of Mmp-3 (1.8-fold, *p* = 0.0352) and Cxcl-12 (1.5-fold, *p* = 0.0136) in prehypertrophic chondrocytes (Figure 4A–F). rmBMP-4 showed an opposite activity on the expression of Pedf, a potent angiostatic factor, in prehypertrophic and hypertrophic chondrocytes with a decreased expression in prehypertrophic chondrocytes (*p* = 0.0478 at 100 ng/mL) and an increased expression in hypertrophic chondrocytes (*p* = 0.0423 and *p* = 0.0392 at 30 ng/mL and 100 ng/mL respectively) (Figure 4I and Figure 5I).

Interestingly, the stimulatory effect of rmGREM-1 on Mmp-3 and Mmp-13 expression and of rmBMP-4 on Rankl mRNA expression was inhibited when osteoblasts were co-stimulated by rmBMP-4 (*p* = 0.0239 and *p* = 0.0077 for Mmp-3 and Mmp-13, respectively) or by rmGREM-1 (Figure 3A–C). Similarly, the respective stimulatory and inhibitory effects of rmGREM-1 and rmBMP-4 on the mRNA expression of Angptl-4 were prevented when osteoblasts were co-stimulated by rmBMP-4 and rmGREM-1, respectively (Figure 3H). In prehypertrophic and hypertrophic chondrocytes, the co-stimulation with rmGREM-1 and rmBMP-4 also inhibited the effect of each of these factors used alone. rmBMP4 blocked the increased expression of Cxcl12 (*p* = 0.0303) and Mmp-3 (*p* = 0.0011) induced by rmGREM-1 in prehypertrophic chondrocytes and hypertrophic chondrocytes, respectively and it tended to blocked that of Mmp-3 (*p* = 0.0854) in prehypertrophic chondrocytes (Figure 4A–F and Figure 5A). The decreased expression of Angptl4 in response to rmBMP-4 was blocked by the co-stimulation with rmGREM-1 in prehypertrophic (*p* = 0.0179) and hypertrophic chondrocytes (*p* = 0.0104) (Figure 4H and Figure 5H).

Together, these results showed that both Grem-1 and its partner BMP-4 could modify the osteochondral junction homeostasis by inducing the expression of a tissue remodeling program in osteoblasts, prehypertrophic, and hypertrophic chondrocytes. Grem-1 rather stimulated catabolic factors, whereas BMP-4-targeted genes associated with osteoclastogenesis and angiogenesis

## 3. Discussion

The pathological remodeling of the osteochondral junction is thought to play a pivotal role in OA. It recapitulates the complex remodeling events that occur during the endochondral ossification process during which cartilage of the growth plate is replaced by bone. Such a process requires coordinated functional interactions between the different cellular partners and should be tightly regulated. Grem-1, through binding to VEGFR2 and inhibiting BMPs, may be one important regulating factors of the osteochondral junction remodeling in OA. As previously reported [28], we observed that chondrocytes in OA cartilage express Grem-1. In addition, we found that Grem-1 positive immunostaining in cartilage increased with disease progression, evaluated by the Mankin score. We also reported an expression of Grem-1 in the OA subchondral bone, which was similarly positively associated with cartilage damage. BMP-2 and BMP-4, which can be targeted by Grem-1, were also expressed in OA cartilage and subchondral bone. Positive immunostaining in cartilage and subchondral bone for both BMPs was positively correlated with cartilage degradation. In agreement, Nakase et al. observed an overexpression of BMP-2 mRNA in severely damaged, as compared to moderately damaged OA cartilage, whereas no expression of BMP-2 mRNA was found in healthy cartilage [39]. The BMP receptors BMPR1a, BMPR1b, and BMPR2 followed the same expression pattern as Grem-1, BMP-2, and BMP-4 in OA cartilage and subchondral bone, characterized by an expression linked to cartilage degradation. In accordance, a positive correlation between the immunohistochemical expression of BMP-2 and BMPR1a in OA cartilage has been already reported [43]. In contrast, although VEGFR2 was detected in OA cartilage, as reported [44,45], no variation according to the degradation of the cartilage was found for VEGFR2 immunostaining.

Our results show that Grem-1, BMP-2, and BMP-4 positive immunostaining was detected in chondrocytes of the deepest part of the cartilage of knee OA patients, as previously described [28,39]. Interestingly, neither BMP-2 mRNA expression nor BMP-2/4 immunostaining was observed in chondrocytes of the deepest layer of the cartilage in healthy samples [39], suggesting that the phenotypic modification of chondrocytes of the deep zone cartilage induces the expression of BMP-2/4. Such chondrocytes display a prehypertrophic phenotype in healthy cartilage and differentiate into hypertrophic chondrocytes in OA. In OA cartilage, hypertrophic chondrocytes were also observed in chondrocyte clusters [46,47]. Moreover, BMP-2 mRNA is expressed in chondrocyte clusters, which are positive for BMP-2/4 and Grem-1 [28,39]. Owing to an in vitro model of progressive hypertrophic differentiation of articular chondrocytes, we recently showed that both Bmp-2 and Bmp-4 mRNA expression increased during the prehypertrophic to hypertrophic differentiation of chondrocytes [42]. Here, we observed that Grem-1 was also overexpressed by hypertrophic chondrocytes, as compared to prehypertrophic ones.

In the subchondral bone, the expression of Grem-1 and its partners was detected in bone cells—osteoblasts and osteocytes—and also in vascular channels. OA subchondral bone is characterized by sclerosis, which results from the phenotypic alteration of osteoblasts and osteocytes [48,49]. The phenotype of OA osteoblasts can be mimicked in vitro by mechanical compression [22]. We show here that compressed osteoblasts expressed and released more Grem-1 and BMP-4 than control uncompressed osteoblasts, whereas the mRNA expression of BMP-2 was not affected. Grem-1 and BMPs are known mechanosensitive genes [28,50]. Moreover, cartilage-derived Grem-1 has been involved in joint damage induced by excessive mechanical loading in a mouse model of post-traumatic OA [28]. However, the subchondral bone of OA patients released more Grem-1 than cartilage from the same patients. Compressed osteoblasts also released more Grem-1 than hypertrophic chondrocytes, suggesting a main role for bone-derived Grem-1. Similarly, OA subchondral bone released more BMP-4 than cartilage from the same patients, whereas BMP-2 was inversely more secreted by OA cartilage. Grem-1 and its partner BMP-4, both released by bone cells more than by cartilage, may thus act together to regulate the pathological remodeling of the osteochondral junction in OA. Our results indeed showed that Grem-1 displays a catabolic activity by stimulating the expression of both Mmp-3 and Mmp-13 expression in chondrocytes and osteoblasts, as previously reported by Chang et al. in chondrocytes [28]. In contrast, BMP-4 rather stimulated the expression of genes associated with angiogenesis and osteoclastogenesis. BMP-4 indeed stimulated the expression of Rankl in osteoblasts and chondrocytes and Ccl-2 in chondrocytes, whereas it decreased that of Angptl-4. Both osteoclastogenesis and angiogenesis are required in endochondral ossification process and are key in OA. Alendronate administration or intraperitoneal injection of osteoprotegerin indeed limited the remodeling of the subchondral bone and the degradation of the cartilage in murine models of post-traumatic OA [51,52]. The number of osteochondral vessels reaching the non-calcified cartilage is linked to the cartilage degradation score and clinical sore of the disease [53]. In addition, the inhibition of VEGF, the main angiogenic factor, by intraperitoneal or intra-articular injection of bevacizumab inhibited the development of OA in rabbit [54].

Grem-1 is a known inhibitor of the BMP pathway. By its interaction with BMPs, among which BMP-4, Grem-1 prevents the binding of BMPs to their receptors. Conversely, this interaction between Grem-1 and BMPs inhibits the BMP-independent activity of Grem-1 [28,55]. Moreover, our results show that Grem-1 and BMP-4 inhibited each other. Especially, the stimulatory effect of BMP-4 on Rankl mRNA expression and its ability to inhibit Angplt-4 mRNA expression were blocked by Grem-1, while the Grem-1-induced expression of Mmp-3 and Mmp-13 was inhibited by BMP-4. Therefore, in addition to their own effects on the expression of tissue remodeling factors by osteoblasts, prehypertrophic, and hypertrophic chondrocytes of the osteochondral junction, the direct interplay between Grem-1 and BMP-4 may also participate in the tight regulation of the complex remodeling processes of the osteochondral junction in OA.

In conclusion, we found that the remodeling of the osteochondral junction in OA is associated with an increased expression of Grem-1 and BMPs. This overexpression of Grem-1 and BMP-4 can be explained by the hypertrophic differentiation of chondrocytes and by the phenotypic change of osteoblasts mediated by mechanical load. Both Grem-1 and BMP-4 may participate in the regulation of the remodeling of the osteochondral junction in OA by modulating angiogenesis, osteoclastogenesis, and matrix degradation.

## 4. Materials and Methods

### 4.1. Collection of Osteoarthritis Human Cartilage and Subchondral Bone

Human OA knee samples (n = 6) were obtained from patients undergoing total knee joint replacement surgery for OA in the Department of Orthopedic Surgery and Traumatology of Saint-Antoine Hospital and in the Maussins-Nollet Clinic (Paris, France). Informed consent was obtained from each patient on the day before arthroplasty. Experiments using human samples have been approved by two French Institutional Review Boards (Comité de protection des personnes Ile de France V, Comité consultatif sur le traitement de l’information en matière de recherche).

Tissue pieces from the middle part of medial and lateral tibial plateaus and femoral condyles were preserved for histological analysis. The remaining cartilage from each joint compartment was separated from the underlining bone and cut into small pieces (1 mm^3^) before anincubation (6 mL/mg wet tissue) in RPMI culture medium supplemented with 100 U/mL penicillin, 100 µg/mL streptomycin, and 4 mM glutamine for 24 h at 37 °C, as described [23]. The same protocol was used for subchondral bone tissue. Conditioned media was then separated from tissues, centrifuged at 3000× *g* for 5 min to removed debris. Conditioned media and tissues were stored at −80 °C until analysis.

### 4.2. Isolation, Culture and Differentiation of Primary Calvaria Mouse Osteoblasts

Osteoblasts were isolated from calvaria of 5–6 days old newborn C57BL/6 mice (Janvier labs), as previously described [21]. Briefly, osteoblasts were cultured 21 days in DMEM/HAM-F12, supplemented with fetal calf serum (10%), penicillin (100 U/mL), streptomycin (100 µg/mL), L-glutamine (4 mM), ascorbic acid (50 µg/mL, Sigma-Aldrich, Saint-Louis, MO, USA). After 10 days of culture, β-glycerophosphate (5 mM, Sigma-Aldrich) was added to the culture medium. At the end of the culture, osteoblasts were washed two times with phosphate buffered saline (PBS) and incubated in DMEM/HAM-F12 without serum for 24 h. Conditioned media were then kept, centrifuged, and stored at −80 °C. Cells were lysed for mRNA extraction.

### 4.3. Isolation, Culture, and Differentiation of Primary Articular Mouse Chondrocytes

Immature articular chondrocytes cells (iMACs) were isolated from femoral heads and knees of 5–6 days old newborn C57BL/6 mice (Janvier labs) and cultured in DMEM culture medium (1 g/L glucose) supplemented with fetal calf serum (10%), penicillin (100 U/mL), streptomycin (100 µg/mL), and L-glutamine (4 mM)) for 7 days, as described [56,57]. Prehypertrophic chondrocytes were obtained by culturing chondrocytes for further 28 days in DMEM/HAM-F12 medium supplemented with fetal calf serum (5%), penicillin (100 U/mL), streptomycin (100 µg/mL) and L-glutamine (4 mM), ascorbic acid (40 µg/mL, Sigma-Aldrich), insulin-transferrin-sodium selenite (1%, Sigma-Aldrich), and triodo-L-thyronine (50 ng/mL, Sigma-Aldrich). Prehypertrophic chondrocytes were cultured for additional 42 days in the latter medium added with β-glycerophosphate (10 mM, Sigma-Aldrich), retinoic acid (100 nM, Sigma-Aldrich), and 1α,25-dihydroxyvitamin D3 (10 nM, Sigma-Aldrich) to obtain hypertrophic chondrocytes [42]. All cultures were performed at 37 °C in a humidified atmosphere of 5% CO_2_/95% air with the exception of prehypertrophic to hypertrophic differentiation step, which was performed at 37 °C in a humidified atmosphere of 3% CO_2_/95% air. At the end of the culture, chondrocytes were washed two times with phosphate buffered saline (PBS) and incubated in DMEM (1 g/L glucose) without serum for 24 h. Conditioned media were then kept, centrifuged, and stored at −80 °C. Cells were lysed for mRNA extraction.

All experiments with murine chondrocytes and osteoblasts were performed according to the protocols approved by French and European ethics committees (Comité d’Ethique en Expérimentation Animale n°5 Charles Darwin de la Région Ile de France).

### 4.4. Compression Experiments

At the end of the culture period, two 12-wells osteoblasts in abundant collagen matrix were collected and placed into a well of a Biopress 6-wells plate (Dunn, Germany), in 1 mL of CO_2_ independent medium D containing 5% FBS, 100 U/mL penicillin, 100 µg/mL streptomycin, 2% of HEPES 1 M and 4 mM glutamine, as described [23]. All of the experiments were performed at 37 °C, in air. An intermittent compression with an air pression of 16.7 Pa at 10% of 1 Hz of frequency was applied for 24 h. The compressive stress was applied to individual samples by the Biopress system (Flexercell International) described by Fermor and colleagues [58], whereas the control osteoblasts were kept in unloaded conditions. After the application of the mechanical regimen, supernatants were collected, centrifuged, and stored at −80 °C for further analysis. The cell pellets were lysed for RNA extraction and were kept at −80 °C. Results are expressed as fold-induction in comparison to controls. Each experiment was carried out in duplicate, and at least nine experiments were performed.

### 4.5. Stimulation of Primary Cultures of Murine Chondrocytes and Osteoblasts

Before treatment, osteoblasts were weaned for 2 h in a serum-free medium DMEM/HAM-F12, supplemented with penicillin (100 U/mL) and streptomycin (100 µg/mL). Prehypertrophic chondrocytes, hypertrophic chondrocytes were weaned for 24 h in a serum-free medium DMEM (1 g/L glucose), containing penicillin (100 U/mL) and streptomycin (100 µg/mL). Cells were stimulated in the same medium by recombinant murine BMP-4 (R&D Systems, Minneapolis, MN, USA) at 30 and 100 ng/mL or Gremlin-1 (R&D Systems) at 100 and 300 ng/mL. After 24-h stimulation, supernatants and total RNA were kept and stored at −80 °C. Pilot studies were performed to establish optimal recombinant protein dosages. Each experiment was carried out in duplicate, and at least five experiments were performed.

### 4.6. Real-Time Quantitative PCR Analysis

Total RNA was extracted using Trizol Reagent (Ambion, Inc., Austin, TX, USA) and following manufacturer’s recommendations. Complementary DNA was obtained from 500 ng of total RNA by reverse transcription (Omniscript RT kit, Qiagen, Courtaboeuf, France). Quantitative RT-PCR was carried out in a Light Cycler 480 (Roche Diagnostics, Meylan, France) using a GoTaq^®^ gPCR Master Mix (Promega Corp., Madison, WI, USA) and specific primers (Supplementary Table S1). Amplification values were normalized with respect to the value obtained for hypoxanthine guanine phosphoribosyl transferase (HPRT), and the expression was determined using the 2^−ΔΔCt^ method.

### 4.7. Protein Quantification and ELISA Assessment of Grem-1, BMP-2, BMP-4

Protein concentration in cell conditioned media was determined using the Bradford assay (Bio-Rad, Hercules, CA, USA). Commercially available ELISA kits were used to determine the concentrations of human and mouse BMP-4 (UscnK Life Science, Wuhan, China), human and mouse Grem-1 (UscnK Life Science, Wuhan, China), human and mouse BMP-2 (R&D Systems), according to the manufacturer’s instructions. The absorbance was measured using a TECAN Infinite M200 plate reader (Tecan, Männedorf, Switzerland).

### 4.8. Histology and Immunohistochemistry

Human tissue samples were fixed in 3.7% paraformaldehyde for 2 days and decalcified in a solution of 14% ethylenediaminetetraacetic acid (EDTA) in distilled water, pH 7.4 for 4 to 6 weeks at 4 °C. Samples were embedded in paraffin wax and serially sectioned (5 µm) (n = 8–15). OA cartilage damage was evaluated on Safranin-O Fast Green stained tissue slices by a Mankin’s score (range 0–14) [59].

Immunohistochemistry was performed with mouse monoclonal antibody to Grem-1 (Abcam, dilution 1:50), BMP-4 (Abcam, dilution 1:50), VEGFR-2 (Abcam, dilution 1:50), BMPR-1a (Abcam, dilution 1:50), BMPR-1b (Abcam, dilution 1:50), BMPR-2 (Abcam, dilution 1:50), as primary antibodies. Enzyme-induced antigen retrieval was performed as follows: 0.2 mg/mL hyaluronidase in PBS, pH 5.5, for 10 min at 37 °C and then 0.1 mg/mL pronase in PBS, pH 7.4, for 20 min at 37 °C. The R.T.U Vectastain kit (Vector) was used for detection, followed by counterstaining with Mayer’s hematoxylin. Irrelevant control antibodies (Dako) were incubated at the same concentration to assess non-specific staining. Preparations were mounted in Eukitt medium.

### 4.9. Image Analysis and Morphometric Analysis

Digital images of magnification views (10×) of whole tissue sections were captured by using an Olympus SC50 camera on an Olympus IX83 microscope. The staining of each protein was quantified according to a semi-quantitative score, on the basis of the intensity and repartition of each staining in the whole joint junction. 0 = no staining, 1 = few staining, 2 = diffuse staining, 3 = intense staining, 4 = high intense staining. Sections were examined blindly in random order by two observers and the scores were averaged to minimize observer bias.

### 4.10. Statistical Analysis

Correlations between Mankin scores and Grem-1, VEGFR-2, BMP-2, BMP-4, BMPR-1a, BMPR-1b, and BMPR-2 staining were established by Spearman’s rank test. The non-parametric Wilcoxon test was used to compare protein levels of BMP-2, BMP-4, and Grem-1 released by human articular cartilage vs. bone in OA patients. The qPCR normalized gene expression levels were compared between compressed and non-compressed osteoblasts, and between prehypertrophic and hypertrophic cells using Wilcoxon test. To analyze the cell response to rmGREM-1 and rmBMP-4, we used Friedman test to determine the dose response effect and adjusted p-value for comparison with the unstimulated group. The analyses mentioned above were performed using GraphPad Prism8 (GraphPad Software Inc., San Diego, CA, USA).

## Data Availability

Not applicable.

## Supplementary Materials

The following supporting information can be downloaded at: https://www.mdpi.com/article/10.3390/ijms23042084/s1.
