# Supplementary material for: Gremlin-1 and BMP-4 Overexpressed in Osteoarthritis Drive an Osteochondral-Remodeling Program in Osteoblasts and Hypertrophic Chondrocytes
Source: Int J Mol Sci. 2022 Feb 14;23(4):2084. doi: 10.3390/ijms23042084 (PMC8874623; doi:10.3390/ijms23042084)
Supplement: Supplementary file 1 [file ijms-23-02084-s001.zip › ijms-1577592-supplementary.pdf]

Table S1. Sequence of mouse primers used for RT-PCR studies.

Gene	Forward primer (5'-3')	Reverse primer (5'-3')
Angptl4	GGGACCTTAACTGTGCCAAG	GAATGGCTACAGGTACCAAACC
Bmp-2	AGATCTGTACCGCAGGCACT	GTTCCTCCACGGCTTCTTC
Bmp-4	GAGGAGTTTCCATCACGAAGA	GCTCTGCCGAGGAGATCA
Bmpr-1a	AGGTCAAAGCTGTTCGGAGA	CTGTACACGGCCCTTTGAAT
Bmpr-1b	CCCTCGGCCCAAGATCCTA	CAACAGGCATTCCAGAGTCATC
Bmpr-2	TATGCAGAATGAACGCAACC	CTGGACATCGAATGCTCAGA
Ccl-2	GATGATCCCAATGAGTAGGCT	TTCTGATCTCATTTGGTTCCGA
Cxcl-12	TGAGAACATGCCTAGATTTACCC	TCATGGCAAGATTCTGGCTTA
Grem-1	TCAAAGCGGGCACATTCAG	AGTAGGAATCGGGTGGTTTGG
Hprt	AGGACCTCTCGAAGTGT	ATTCAAATCCCTGAAGTACTCAT
Mmp3	TGAAAATGAAGGGTCTTCCGG	GCAGAAGCTCCATACCAGCA
Mmp13	GATGGCACTGCTGACATCAT	TGTAGCCTTTGGAACTGCTT
Pedf	GAGGGAAATGCCCAGTGC	ATCAGAGTCCAAGCCGTATC
Rankl	TGAAGACACACTACCTGACTCCTG	CCACAATGTGTTGCAGTTCC
Tsp-1	GCTGGCAATGCACCTTCTAA	TAAAGGCCGAGTGCTGAC
Vegf	CTGCTGTGGACTTGTGTTG	ATGGGTTTGTCGTGTTTCT
Vegfr-2	AGCACCTCTCTCGTGATTTCC	AGTAAAAGCAGGGAGTCTGTGG

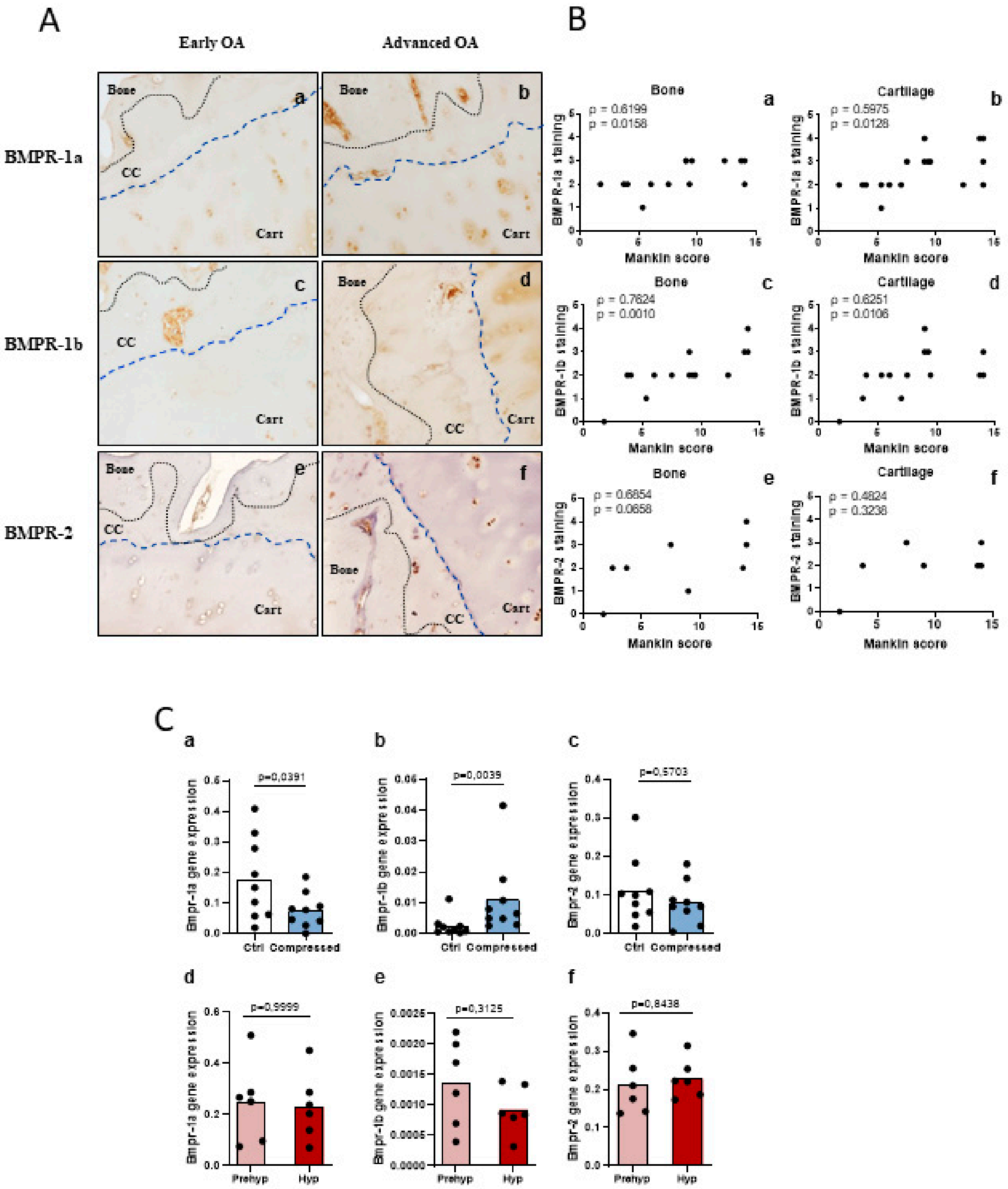

Figure S1. Expression of BMPRs in human OA tissues, compressed osteoblasts, prehypertrophic and hypertrophic chondrocytes. Panel A). Immunohistochemical staining of BMPR-1a (a, b), BMPR-1b (c, d) and BMPR-2 BMP-2 (e, f) at bone/cartilage interface of human OA knee samples. Panel B). Correlations between OA score, evaluated by the Mankin score, and the expression of BMPR-1a (a, b), BMPR-1b (c, d) and BMPR-2 (e, f) in bone (a, c and e) and cartilage (b, d and f) (n=8-17). Panel C.

mRNA expression of BMPR-1a, BMPR-1b and BMPR-2 in compressed and control osteoblasts (n=9-12) (a-c) and in prehypertrophic and hypertrophic chondrocytes (n=6) (e-f) was determined. Cartilage and bone are delimited by black dotted lines, and cartilage and calcified cartilage are delimited by blue dotted lines. Cart: cartilage, CC: calcified cartilage. Scale bars = 50 µm. Pictures are representative of 17 tissue samples from 6 OA patients. Bars indicate the mean expression levels.
